# Microbial production of ectoine and hydroxyectoine as high-value chemicals

**DOI:** 10.1186/s12934-021-01567-6

**Published:** 2021-03-26

**Authors:** Mengshuang Liu, Hui Liu, Meng Shi, Mingyue Jiang, Lingling Li, Yanning Zheng

**Affiliations:** 1 State Key Laboratory of Microbial Resources, Institute of Microbiology, Chinese Academy of Sciences, No. 1 Beichen West Road, Chaoyang District, Beijing, 100101 China; 2 CAS Key Laboratory of Bio-Based Materials, Qingdao Institute of Bioenergy and Bioprocess Technology, Chinese Academy of Sciences, Qingdao, China; 3 University of Chinese Academy of Sciences, Beijing, China; 4 Department of Microbiology, University of Washington, Seattle, USA

**Keywords:** Metabolic engineering, Halophiles, Model microorganisms, Ectoine, Hydroxyectoine

## Abstract

Ectoine and hydroxyectoine as typical representatives of compatible solutes are not only essential for extremophiles to survive in extreme environments, but also widely used in cosmetic and medical industries. Ectoine was traditionally produced by *Halomonas elongata* through a “bacterial milking” process, of which the marked feature is using a high-salt medium to stimulate ectoine biosynthesis and then excreting ectoine into a low-salt medium by osmotic shock. The optimal hydroxyectoine production was achieved by optimizing the fermentation process of *Halomonas salina*. However, high-salinity broth exacerbates the corrosion to fermenters, and more importantly, brings a big challenge to the subsequent wastewater treatment. Therefore, increasing attention has been paid to reducing the salinity of the fermentation broth but without a sacrifice of ectoine/hydroxyectoine production. With the fast development of functional genomics and synthetic biology, quite a lot of progress on the bioproduction of ectoine/hydroxyectoine has been achieved in recent years. The importation and expression of an ectoine producing pathway in a non-halophilic chassis has so far achieved the highest titer of ectoine (~ 65 g/L), while rational flux-tuning of halophilic chassis represents a promising strategy for the next-generation of ectoine industrial production. However, efficient conversion of ectoine to hydroxyectoine, which could benefit from a clearer understanding of the ectoine hydroxylase, is still a challenge to date.

## Background

Increased environmental osmolarity will trigger the outflow of intracellular water across the cell membrane, resulting in the loss of turgor and then the shrinkage of cell. The overcrowding of biomacromolecules lowers their diffusion rates in cytoplasm, affects normal cellular physiological activities, and ultimately threatens cell survival [1, 2]. To avoid the detrimental effects caused by the high environmental osmolarity, some halophilic microorganisms are able to balance the intra- and extracellular osmotic pressure by accumulating large amounts of specific small organic molecules called compatible solutes, whose massive accumulation does not disturb normal cellular process [3, 4]. Compatible solutes, mainly including sugars (sucrose, trehalose and so on), polyols (glycerol, glucosyl-glycerol, mannosyl-glycerol, arabitol, sorbitol, mannitol and so on), amino acids and their derivatives (proline, glycine betaine, ectoine, hydroxyectoine and so on), are widely distributed in nature [5, 6].

As typical representatives of compatible solutes, ectoine (1,4,5,6-tetrahydro-2-methyl-4-pyrimidinecarboxylic acid) is a cyclic derivative of aspartate while hydroxyectoine (1,4,5,6-tetrahydro-2-methyl-5-hydroxy-4-pyrimidinecarboxylic acid) is a hydroxylated derivative of ectoine [7, 8]. In addition to acting as osmotic protective agents, ectoine and hydroxyectoine are also excellent biofunctional stabilizers, skin protectors and potential drugs for diseases, such as Alzheimer’s and rhinoconjunctivitis symptom [9–14]. Ectoine has become a high-demand product because of its wide application in biotechnology, cosmetics and medicine, and also represents a multibillion-dollar market, with an annual market demanding of about 15,000 tons and a retail price of about 1000 USD/kg [15]. The current process for hydroxyectoine bioproduction can only provide a mixture of ectoine and hydroxyectoine, which are chemically similar compounds. Given it is not easy to isolate hydroxyectoine from the mixture of ectoine and hydroxyectoine, bioproduction of hydroxyectoine on a large scale was economically unattractive. However, hydroxyectoine has a higher glass transition temperature than ectoine, making it be a better desiccation protectant [16, 17]. Therefore, increasing attention has been paid to the microbial production of ectoine/hydroxyectoine in recent years.

**Fig. 1:**
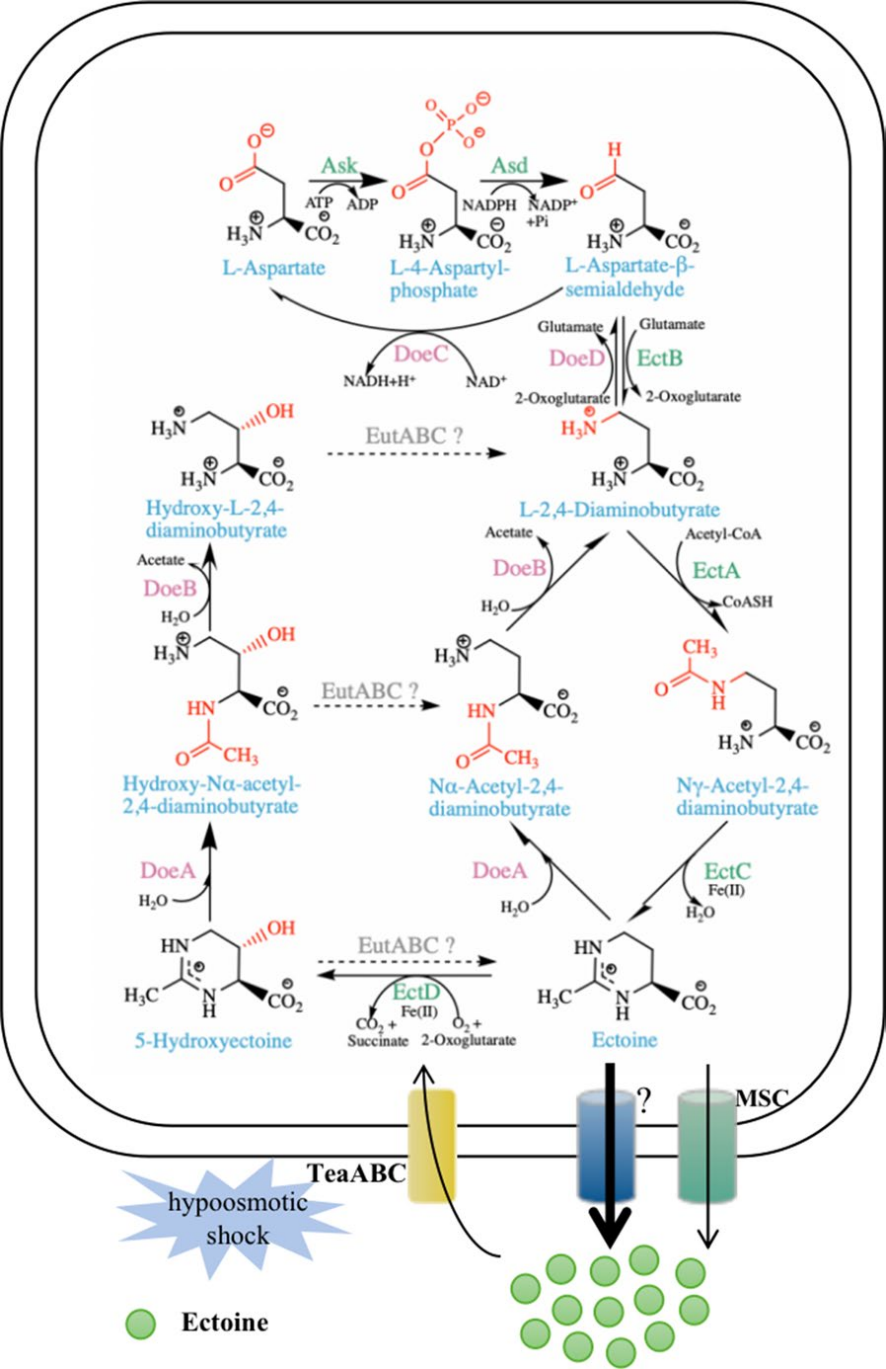
Ectoine biosynthesis and biodegradation pathways. *Ask* l-aspartate kinase, *Asd* l-aspartate-β-semialdehyde-dehydrogenase, *EctB* l-2,4-diaminobutyrate aminotransferase, *EctA* l-2,4-diaminobutyrate -acetyltransferase, *EctC* ectoine synthase, *EctD* ectoine hydroxylase, *EutABC?* hypothesized enzymes for hydroxyectoine degradation, *DoeA* ectoine hydrolase, *DoeB* *N-*α-acetyl-l-2,4-diaminobutyric acid deacetylase, *DoeD* diaminobutyric acid transaminase, *DoeC* Aspartate-semialdehyde dehydrogenase, *TeaABC* TRAP transporter for ectoine, *?* unknown efflux system for ectoine, *MSC* mechanosensitive channels

The biosynthesis of ectoine from aspartate is carried out by sequential catalysis of five enzymes, namely, l-aspartate kinase (Ask), l-aspartate-β-semialdehyde dehydrogenase (Asd), l-2,4-diaminobutyrate aminotransferase (EctB), l-2,4-diaminobutyrate acetyltransferase (EctA) and ectoine synthase (EctC) [18, 19]. Ectoine hydroxylase (EctD), which catalyzes the conversion of ectoine to hydroxyectoine through a regio- and stereo-specific hydroxylation reaction, has also been found in many native ectoine producers [8, 20, 21]. Genes encoding EctB, EctA and EctC are usually present as a gene cluster (*ectABC*), sometimes with *ectD* in the context [17]. The expression of *ectABC(D)* is triggered by an increase of environmental salinity [22]. Disruption of *ectABC(D)* will make halophiles sensitive to high-salinity levels, indicating that ectoine and hydroxyectoine play important roles for microorganisms in adaptation to high-salinity environment [17, 23]. In addition to being compatible solutes, ectoine and hydroxyectoine are able to be degraded by some microorganisms for carbon and nitrogen sources [24, 25]. Based on the genome analysis of *H. elongata* DSM 2581T, Schwibbert et al*.* described the ectoine degradation pathway for the first time [26]. Their proposed Doe pathway converts ectoine back to aspartate with a different set of enzymes, namely, ectoine hydrolase (DoeA), *N*-α-acetyl-l-2,4-diaminobutyric acid deacetylase (DoeB), diaminobutyric acid transaminase (DoeD) and aspartate-semialdehyde dehydrogenase (DoeC) (Fig. 1). The pathway for the catabolism of hydroxyectoine has been proposed in *Ruegeria pomeroyi*. EutA, EutB and EutC are probably responsible for the conversion of hydroxyectoine into ectoine, which is further degraded into aspartate using the above-mentioned Doe pathway [27]. However, Christopher et al. found that the EutD (DoeA)/EutE (DoeB) from *R. pomeroyi* could degrade hydroxyectoine into hydroxy-l-2,4-diaminobutyrate (hydroxy-DABA) via hydroxy-*N*α-acetyl-l-2,4-diaminobutyrate (hydroxy-*N*α-ADABA) as an intermediate [28], implying that a different pathway for hydroxyectoine degradation may exist (Fig. 1).

Industrially, ectoine is produced by *Halomonas elongata* through a “bacterial milking” process [29]. In this process, high-salt medium is used to stimulate ectoine biosynthesis, and then the bacterial cells are shocked by low-salt medium to rapidly release the synthesized ectoine into the medium. Hydroxyectoine production can be improved by increasing medium salinity and culture temperature [30]. However, high-salt medium exacerbates the corrosion of conventional steel fermenter and increases the difficulty of wastewater treatment [31–33], all of which brings extra cost of ectoine/hydroxyectoine bioproduction. Given the disadvantages caused by high-salt medium, researchers have made efforts to lower the salinity normally required for efficient biosynthesis of ectoine/hydroxyectoine (Table 1).

**Table 1:** Microbial production of ectoine and hydroxyectoine

Strain	Salinity^a^ (M)	Titer (g/L)	Productivity (g/L/h)	Yield (g/g)	Carbon source	References
Ectoine						
*H. elongata* DSM142	2.57	7.4	0.22	0.11	Glucose	[30]
*B. epidermis* DSM 20659	1.42	8	0.08	0.05	Monosodium glutamate	[31]
*H. salina* DSM5928	1.81	14.86	0.32	0.14	Monosodium glutamate	[33]
*H. salina* BCRC17875	2.47	13.96	0.29	NA	Monosodium glutamate	[42]
*H. hydrothermalis* Y2	1.14	10.5	0.22	0.21^b^	Monosodium glutamate and glucose	[46]
*E. coli* DH5α (pASK-*ectABC*)	0.08	6	0.04	NA	Glucose	[32]
*E. coli* BW25113	0.4	25.1	1.04	NA	Glycerol and aspartate	[51]
*E. coli* ECT05	0.03	25.1	0.84	0.11	Glucose	[34]
*E. coli* DH5α (pASK_ectABCD_m_)	0.22	1.7	NA	0.36	Glycerol	[59]
*C. glutamicum* Ect-2	0.03	4.5	0.28	NA	Glucose	[66]
*C. glutamicum* Ecto5	0.01	22	0.32	0.16	Glucose	[68]
*C. glutamicum ectABC*^*opt*^	0.03	65	1.16	0.19^c^	Glucose and molasses	[35]
Hydroxyectoine						
*H. salina* BCRC17875	0.56	2.9	NA	NA	Monosodium glutamate	[43]
*H. polymorpha* ALU3/EctBACD	0	2.8	0.02	NA	MeOH and sorbitol	[72]
*E. coli* FF4169 (pMP41)	0.4	2.13	0.09	NA	Glucose and ectoine	[57]
*E. coli* DH5α (pASK_ectABCDask)	0.26	1.6	NA	0.34	Glycerol	[59]

*NA* not available

^a^The salinity is shown as the combined concentration of sodium and potassium salts in the medium

^b^The yield here is calculated with monosodium glutamate

^c^The yield here is calculated with glucose and molasses

Synthetic biology, through which the gene regulation can be eliminated to artificially control the gene expression, plays a key role in the development of high-value chemicals, including ectoine/hydroxyectoine. Quite a lot of advances in improving ectoine/hydroxyectoine production under low-salinity conditions and in enhancing the exportation of ectoine/hydroxyectoine have been achieved through the synthetic biology. Given that *E. coli* and *Corynebacterium glutamicum* normally grow at low-salinity levels, they have recently been used as bacterial chassis for ectoine/hydroxyectoine production [34, 35]. Though *E. coli* and *C. glutamicum* do not naturally accumulate ectoine and hydroxyectoine, they can be endowed with the ability to produce ectoine/hydroxyectoine through synthetic biology. More recently, enhanced ectoine production has been achieved by rational flux-tuning in a chromosomally engineered *H. bluephagenesis*, which allows open unsterile and continuous growth conditions, providing a promising bacterial chassis for next generation of industrial biotechnology [36].

## Microbial production of ectoine/hydroxyectoine in halophilic bacteria

### Halomonas elongata

Ectoine was initially produced by the “bacterial milking” process in a non-engineered halophilic bacterium *H. elongata*. By adopting the “bacterial milking” process, a final ectoine titer of 7.4 g/L was achieved after repeatedly performing this process at least nine times, with a productivity of 0.22 g/L/h. Mechanosensitive channels (MSC), which are responsible for the excretion of compatible solutes in many halophilic microorganisms when suffering hypoosmotic shock, only make a small contribution to the ectoine excretion in *H. elongata* [37, 38]. Therefore, *H. elongata* should have an efflux system either as a universal compatible solute transporter or as a specific ectoine exporter [38]. By deleting the Trap-TeaABC transporter for ectoine uptake and disrupting the Doe pathway for ectoine degradation, Kunte’s research group developed a “super-leaky” *H. elongata* mutant, which is capable of exporting synthesized ectoine to the medium without hypoosmotic shock [26, 29, 39]. The above-mentioned techniques make an annual production of ectoine in tons possible, but high-salt media are still required.

When *H. elongata* was grown in a synthetic medium containing 4.27 M NaCl at 40 °C, it produced more hydroxyectoine than ectoine. However, it is economically infeasible to isolate hydroxyectoine from the mixture of ectoine and hydroxyectoine because of their high chemical similarity, making the industrial production of hydroxyectoine unattractive [6, 9]. Therefore, *H. elongata* was engineered to improve the conversion of ectoine to hydroxyectoine by heterogenous expression of the ectoine hydroxylase gene (*thpD*) from *Streptomyces chrysomallus*, the engineered strain was capable of converting all ectoine to hydroxyectoine [40]. But unfortunately, the medium salinity used in the study is still high (1.7 M).

### Brevibacterium epidermis

*Brevibacterium epidermis*, which naturally produces ectoine, can tolerate salinities as high as 2 M NaCl. Its ectoine production correlates positively with the NaCl concentrations up to 1 M. In addition, *B. epidermis* has no *ectD* gene, so it is unable to synthesize hydroxyectoine, which is a common by-product in ectoine biosynthesis [24]. *B. epidermis* has been used for ectoine production with a salt level of 1.42 M. The fed-batch fermentation of this bacterium using monosodium glutamate as substrate achieved an ectoine titer of 8 g/L, a productivity of 0.08 g/L/h, and a yield of 0.05 g/g monosodium glutamate [31]. *B. epidermis* has several advantages in ectoine production when compared with *H. elongata*. Firstly, it uses a lower salt level (~ 1.42 M) than *H. elongata* (~ 2.57 M NaCl). Secondly, it can avoid a further consumption of ectoine and simplify the downstream ectoine recovery process, as it is naturally unable to make by-product hydroxyectoine. However, relatively high salinity and hypoosmotic shock are also required for efficient ectoine production by *B. epidermis*.

### Halomonas salina

*Halomonas salina* DSM 5928 can synthesize and secrete ectoine at a constant extracellular osmotic pressure [41]. The NaCl concentrations ranging from 0.5 M to 2 M do not affect the final ectoine production, although a lower salt level contributes to a better excretion of ectoine. Therefore, *H. salina* exhibits advantages over *H. elongata* and *B. epidermis* in terms of medium-salt production of ectoine. When using monosodium glutamate as the carbon and nitrogen source in a batch fermentation, *H. salina* DSM 5928 produced 6.9 g/L ectoine at a salt level of 0.5 M NaCl, with a productivity of 0.33 g/L/h and a secretion rate of 61.6% [41]. To achieve a better ectoine production, Lang et al*.* adopted a two-phase strategy. They firstly grew bacterial cells in a batch fermentation, then harvested the cells, and finally used phosphate-limited non-growing cells for ectoine production. By separating cell growth from ectoine biosynthesis, 14.86 g/L ectoine was produced by *H. salina* DSM 5928, with a productivity of 0.32 g/L/h and a yield of 0.14 g/g monosodium glutamate. In addition, about 79% of ectoine was excreted into the medium [33].

Benefiting from the natural property of ectoine excretion, *H. salina* DSM 5928 can achieve a high ectoine production without hypoosmotic shock. Simultaneous ectoine biosynthesis and excretion not only simplifies the process of ectoine production, but also reduces the intracellular ectoine consumption by preventing ectoine from entering the Doe catabolic pathway. Additionally, dissolved oxygen is an important factor for efficient ectoine production. However, high cell density fermentation, which is always adopted for enhanced ectoine production by *H. elongata* and *B. epidermis*, can cause an undersupply of dissolved oxygen. An alternative strategy for reducing the consumption of oxygen is to use element-limited non-growing cells. Indeed, two-fold more ectoine was produced by non-growing *H. salina* DSM 5928 cells when compared with its growing cells.

*Halomonas salina* BCRC 17875 has also been used for the production of ectoine/hydroxyectoine [42, 43]. By optimizing the agitation speed and medium composition, 13.96 g/L ectoine was finally obtained using monosodium glutamate as substrate, with a productivity of 0.29 g/L/h [42]. In addition, 2.9 g/L hydroxyectoine was achieved in medium supplemented with 50 mM α-ketoglutarate and 1 mM iron, representing the highest hydroxyectoine production to date [43].

### Halomonas hydrothermalis

*Halomonas hydrothermalis*, originally isolated from the alkaline pulp mill wastewater, grows well at salt levels ranging from 0 to 3 M NaCl [44]. It expresses both ectoine biosynthesis pathway and ectoine degradation pathway, with the *ectABC* and *doeABXCD* gene clusters responsible for ectoine biosynthesis and ectoine degradation, respectively. Moreover, it also has an *ectD* gene locating remotely from the *ectABC* gene cluster [45]. By deleting the *ectD* and *doeA* genes, the ectoine production was increased from 5.5 to 7.2 g/L in a batch fermentation [46]. By further knocking out the *mrp* gene, whose protein product is a Na^+^/H^+^ antiporter and plays an important role in pH and osmotic homeostasis, the optimal NaCl concentration for ectoine biosynthesis dropped from 1.7 to 1.4 M [47]. Finally, the engineered *H. hydrothermalis* Y2/*ΔectD*/*ΔdoeA*/*Δmrp* grown in the fermentation broth supplemented with ~ 1 M NaCl accumulated up to 10.5 g/L of ectoine in a fed-batch fermentation, with a productivity of 0.22 g/L/h, a yield of 0.21 g/g monosodium glutamate and an ectoine excretion rate of 62%.

Though the engineered strain *H. hydrothermalis* Y2/*ΔectD*/*ΔdoeA*/*Δmrp* produced less ectoine than *H. salina*, the salinity level required for maximal ectoine production can be further lowered by limiting the Na^+^ outflow, representing a new strategy for the low-salt production of compatible solutes. However, ectoine production decreased by ~ 20% in the late stage of *H. hydrothermalis* Y2/*ΔectD*/*ΔdoeA* fermentation, indicating that *H. hydrothermalis* may still have some unknown ectoine degradation pathways. However, a further investigation is needed to verify this assumption. The TRAP-TeaABC transporter is responsible for ectoine uptake, and the deletion of *teaC* and *teaBC* genes in *H. elongata* can greatly enhance the excretion of ectoine, yielding a “super leaky” mutant. It suggests the function of TeaABC is probably to transport the extracellular ectoine into the cell and also participate in the negative regulation of ectoine biosynthesis [39]. Similar to *H. elongata*, the *teaABCD* gene cluster also exists in the genome of *H. hydrothermalis* [45]. Therefore, the deletion of *teaABCD* gene cluster is a promising strategy to further improve the production of ectoine in *H. hydrothermalis*.

## Microbial production of ectoine/hydroxyectoine in non-natural host strains

### Escherichia coli

*Escherichia coli* is widely used as host for heterologous protein expression due to its long history of laboratory culture, clear genetic background, ease of manipulation, and good compatibility with heterologous proteins [48]. In addition, *E. coli* does not have neither *ectD* gene nor *doeABCD* genes, making it unable to convert or degrade the synthesized ectoine [49]. Therefore, *E. coli* has been engineered for heterologous production of ectoine with low-salt medium. Louis et al. expressed the *ectABC* gene cluster from *Marinococcus halophilus* in *E. coli* XL1-Blue for the production of ectoine, representing the first successful attempt of introducing ectoine biosynthetic pathway into *E. coli* [50]. The synthesized ectoine increased the tolerance of the engineered *E. coli* to the hyperosmotic stress (up to 0.85 M NaCl). Subsequently, Schubert et al. introduced a *tet* promoter controlled *ectABC* from the halophilic bacterium *Chromohalobacter salexigens* into the *E. coli* DH5α [32]. Under the control of the inducible *tet* promoter, the cell growth phase of *E. coli* was separated from its ectoine biosynthesis phase. About 6 g/L ectoine was excreted by the engineered *E. coli* DH5α using glucose as substrate, with an ectoine productivity of only 0.04 g/L/h, which is much lower than that achieved in halophilic bacteria. Thus, ectoine production still need to be improved by employing inducible promoters with stronger and more stable expression of the ectoine biosynthetic pathway. An arabinose-inducible promoter was subsequently selected to drive the expression of *H. elongata ectABC* gene cluster in *E. coli* BW25113 [51]. The engineered *E. coli* achieved an ectoine titer of up to 25.1 g/L and an ectoine productivity of 1.04 g/L/h by whole-cell catalysis when the bacterial cells were cultured with glycerol and aspartate as substrates. The whole-cell biocatalysis represents an alternative strategy to improve the ectoine bioproduction in engineered strain.

**Fig. 2:**
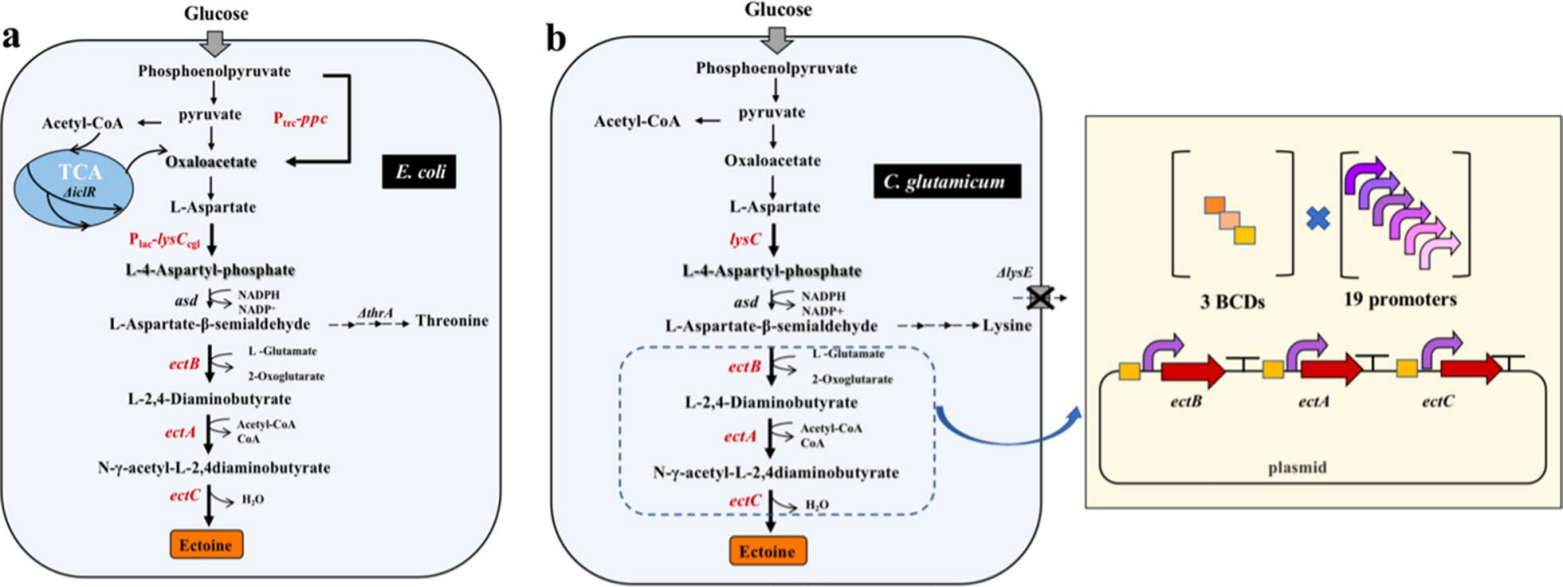
Metabolic engineering modification strategy for ectoine production by *E. coli* and *C. glutamicum.* **a** Metabolic engineering modification strategy for ectoine production by *E. coli*. **b** Metabolic engineering modification strategy for ectoine production by *C. glutamicum*. Control element library for transcriptionally balanced expression of the ectoine synthesizes related genes. The library was constructed from 3 BCDs (bicistronic designed elements), 19 promoters and the terminator rrnBT1T2

*Escherichia coli* W3110 has been successfully applied to synthesize aspartate-derived products, such as l-threonine and 3-aminopropionic acid, implying its potential in ectoine biosynthesis [52, 53]. Ning et al. successfully introduced the *ectABC* gene cluster from *H. elongata* into *E. coli* W3110 and uncoupled the gene expression from osmotic pressure induction by using a strong *trc* promoter [34]. To further improve the ectoine production, they made efforts to provide the bacterial cells with sufficient precursor aspartate-β-semialdehyde (ASA) and then increase the metabolic flux from ASA to ectoine. Thus, the *thrA* gene coding for a bifunctional aspartate kinase/homoserine dehydrogenase was deleted to block the l-threonine branch, and the *lysC* gene encoding the feedback inhibition resistant aspartokinase was overexpressed to complement the aspartokinase activity impaired by *thrA* deletion. The native promoter of *ppc* gene for phosphoenolpyruvate carboxylase was also replaced with a stronger *trc* promoter, and the *iclR* gene, whose protein product is responsible for the transcriptional repression of the glyoxylate branch, was deleted to increase the supply of oxaloacetate. Finally, the engineered *E. coli* ECT05 accumulated 25.1 g/L ectoine in a fed-batch fermentation using glucose as substrate, with a productivity of 0.84 g/L/h and a yield of 0.11 g/g glucose. Altogether, a high ectoine production has been achieved at a salinity of as low as 0.03 M through the metabolic engineering of *E. coli* (Fig. 2a).

Given that EctB is a rate-limiting enzyme for ectoine biosynthesis, Chen et al. established a high-throughput screening method in *E. coli* to screen *He*EctB (*ectB* from *H. elongata*) variants with increased activities by engineering the regulatory protein AraC to use ectoine as its effector [54]. The expression of the quantitative reporter GFP is positively correlated with the ectoine production. Finally, a mutant (D180V/F320Y/Q325R) with obviously improved ectoine production was obtained by combining the directed evolution and high-throughput screening. More recently, the crystal structure of *Cs*EctB (*ectB* from *C. salexigens* DSM 3043) has been determined. When structurally compared with the *Cs*EctB, all of the three point mutations (D180V/F320Y/Q325R) of *He*EctB variant are located on the protein surface but not the active center [55]. These mutations may be responsible for the increased structural flexibility by affecting the electrostatic interaction (D180V), the hydrophobic patch (F320Y) and the surface charge alteration (Q325R) [55]. The solved crystal structure of EctB will definitely contribute to the rational design of this rate-limiting enzyme for more efficient production of ectoine, though a good balance between its activity and stability should be taken into consideration.

*Escherichia coli* has also been used for the production of hydroxyectoine. Under the control of its salt-inducible native promoter, the whole *ectABCD-ask* gene cluster from *P. stutzeri* DSM5190 was successfully expressed in *E. coli* DH5α. The engineered strain requires a medium supplemented with 0.34 M NaCl for efficient hydroxyectoine production [56]. Subsequently, Czech et al. expressed *ectD* from *P. stutzeri* A1501 in *E. coli* FF4169 (defective in the trehalose synthesis) under the control of the inducible *tet* promoter. The added ectoine, which was imported into the cells with the help of osmotic stress inducible ProP and ProU transporters, was completely converted to hydroxyectoine in a shake-flask cultivation. Finally, a hydroxyectoine titer of 2.13 g/L and a productivity of 0.09 g/L/h were obtained using a medium supplemented with 0.4 M salt [57]. However, it is still not economical to directly convert ectoine to hydroxyectoine in bulk because of the high price of ectoine.

In addition, the ectoine/hydroxyectoine biosynthetic gene clusters are usually obtained from halophilic microorganisms. When these gene clusters are expressed in *E. coli*, the activities of their gene products (enzymes) could be greatly impaired [58]. Therefore, Bethlehem et al. expressed the ectoine/hydroxyectoine biosynthetic genes from a non-halophilic bacterium (*Acidiphilium cryptum*) in *E. coli* [59]. Specifically, *E. coli* carrying pASK_ectABCD_m_ and pASK_ectABCDask were used for the production of ectoine and hydroxyectoine, respectively. The pASK_ectABCD_m_ expresses EctABC and an inactivated EctD, while the pASK_ectABCDask expresses EctABCD and an additional aspartokinase. In a shake-flask cultivation using glycerol as substrate, an ectoine titer of 1.7 g/L and a productivity of 0.36 g/g glycerol were achieved with almost no detectable byproducts. In contrast to the ectoine production, a hydroxyectoine titer of 1.6 g/L and a productivity of 0.34 g/g glycerol were obtained and only 4% ectoine was detected as a byproduct, representing a promising strategy for the production of hydroxyectoine economically.

### Corynebacterium glutamicum

*Corynebacterium glutamicum*, which has a long history in the production of amino acids, especially glutamate and lysine, is another microbial chassis commonly used [60–62]. The feedback inhibition resistant aspartokinase has been developed to provide lysine production with plenty of ASA precursor [63], which is also an important precursor compound for the biosynthesis of ectoine. Given the large amount of lysine produced by *C. glutamicum*, one might expect *C. glutamicum* is also an ideal host for the heterologous production of ectoine. In addition, *C. glutamicum* has no ectoine degradation pathway just like *E. coli*, capable of avoiding the consumption of ectoine and the formation of byproduct [64].

The lysine-producing strain *C. glutamicum* LYS-1 has been selected for the ectoine biosynthesis because it expresses a feedback inhibition resistant aspartokinase (LysC^T311I^) and thus can synthesize large amount of ASA [65]. Becker et al. expressed the *ectABCD* gene cluster from *Pseudomonas stutzeri* in the genome of *C. glutamicum* LYS-1, with *ectABCD* under the control of a strong constitutive promoter *tuf* [66]. The *ddh* gene coding for diaminopimelate dehydrogenase, which diverts most of the metabolic flux into the dehydrogenase branch of the lysine biosynthetic pathway when the ammonia level is high, was selected as the integration region to decrease the carbon flux from ASA to lysine. However, lysine still can be detected as a by-product. To further prevent the excretion of lysine, the *lysE* gene encoding the lysine exporter LysE was knocked out. The deletion of *lysE* gene blocked the excretion of lysine but had no effect on the excretion of ectoine. Finally, the engineered *C. glutamicum* ECT-2 grown in low-salt medium (0.03 M salt) accumulated 4.5 g/L ectoine in a fed-batch fermentation, with a productivity of 0.28 g/L/h. Given that only 0.4 g/L hydroxyectoine was produced by *C. glutamicum* ECT-2 strain, the succinylase branch might divert a part of the carbon flux into by-product lysine, and the heterologous ectoine transporters might also affect the biosynthetic efficiency of ectoine in the engineered *C. glutamicum*.

In addition to expressing a previously described feedback inhibition resistant aspartokinase LysC^T311I^, another lysine-producing strain *C. glutamicum* DM1729 has a variant pyruvate carboxylase Pyc^P458S^ and a variant homoserine dehydrogenase Hom^V59A^, which exhibits a higher activity than the wild-type pyruvate carboxylase Pyc and a lower activity than the wild-type homoserine dehydrogenase Hom, respectively. The enhanced activity of Pyc^P458S^ makes more oxaloacetate precursor available for lysine biosynthesis, while the impaired activity of Hom^V59A^ limits the conversion of ASA to threonine and methionine [67]. *C. glutamicum* DM1729 could be a promising microbial chassis for enhanced production of ectoine since it has already achieved a good lysine production, which shares a lot of metabolic fluxes with ectoine biosynthesis. Therefore, Pérez-García et al. expressed the ectoine biosynthetic pathway in *C. glutamicum* DM1729 for the production of ectoine. The host genes *sugR* and *ldhA*-coding for a regulator and lactate dehydrogenase, respectively-were further deleted to improve the metabolism of glucose and avoid the formation of lactic acid [68]. The engineered strain *C. glutamicum* Ecto5 (DM1729*ΔsugRΔldhA*) grown in low-salt medium (0.01 M salt) accumulated 22 g/L ectoine in a fed-batch fermentation, with a productivity of 0.32 g/L/h and a yield of 0.16 g/g glucose, respectively. However, a large amount of by-product lysine still can be detected in the fermentation broth. Though attempt has been made to avoid the lysine efflux, the deletion of *lysE* gene results in dramatically decreased ectoine production and obviously impaired cell growth. Unlike *C. glutamicum* ECT-2, a further deletion of *lysE* in *C. glutamicum* Ecto5 may cause the metabolic imbalance of the bacterial cell.

More recently, Giesselmann et al. used *C. glutamicum lysC*^*fbr*^ as starting strain for the heterologous production of ectoine [35]. A series of genetic modification on this lysine-producing strain was carried out to further improve the ectoine biosynthesis (Fig. 2b). Firstly, the *lysE* gene was deleted to block the excretion of lysine. Then, a transcription balance strategy was adopted to systematically optimize the ectoine biosynthetic pathway cloned from *P. stutzeri*, resulting in higher production levels of ectoine. Unlike the previous strategy of using one promoter to control the expression of *ectABC* genes, 19 synthetic promoters and 3 bicistronic linkers were employed to coordinate the co-expression of *ectA*, *ectB* and *ectC* by creating an expression library with 185,193 variants. The engineered *C. glutamicum ectABC*^*opt*^ obtained by high-throughput screening of transformants produced 65 g/L ectoine in a fed-batch fermentation fed with glucose-molasses medium (0.03 M salt), representing the highest ectoine titer to date. A good productivity of 1.16 g/L/h and a good yield of 0.19 g/g glucose-molasses were also achieved in this fermentation process, with just a small amount of trehalose detected as by-product (3 g/L). In addition, by comparing *C. glutamicum ectABC*^*opt*^ with a part of other mutants in the library, the expression ratio of *ectA*/*ectB* was found to be of great importance in determining the pathway flux, with the best ectoine producer exhibiting an EctA:EctB ratio of 1:3.1 by proteome analysis. This valuable finding facilitates the subsequent development of efficient chassis cells for further enhanced ectoine production.

### Hansenula polymorpha

*Hansenula polymorpha*, which is a methylotrophic yeast, has been well developed for the expression of heterologous proteins, and it has also been used as a whole-cell biocatalyst [69–71]. In order to produce hydroxyectoine, synthetic ORFs encoding EctA, EctB, EctC and EctD from *H. elongata* were inserted into the genome of *H. polymorpha*. When grown in the medium supplemented with methanol and sorbitol as carbon source, the engineered *H. polymorpha* produced 2.8 g/L hydroxyectoine, with only 0.044 g/L ectoine as a by-product [72]. Moreover, almost no salt is required for efficient production of hydroxyectoine in *H. polymorpha*, exhibiting advantages over other microbial chassis in terms of low-salt production of hydroxyectoine. However, the long fermentation time (144 h) undoubtedly increases the cost of hydroxyectoine production, posing a problem to the industrial production of hydroxyectoine in *H. polymorpha*.

## Microbial production of ectoine/hydroxyectoine based on next generation industrial biotechnology

With increasing stress from environmental pollution, growing attention is being paid to the microbial production of chemicals in a more environment-friendly manner. The “next generation industrial biotechnology” based on moderately halophilic bacteria has advantages over the current industrial biotechnology. Firstly, no strict sterilization is required for the media, decreasing the energy consumption and process complexity. Secondly, cheap plastic or even cement fermentation tanks can be used for mass production, avoiding the corrosion to the traditional steel fermentation tanks and reducing the production cost. Thirdly, sea water can be used directly with a simple processing, saving a lot of valuable fresh water.

**Fig. 3:**
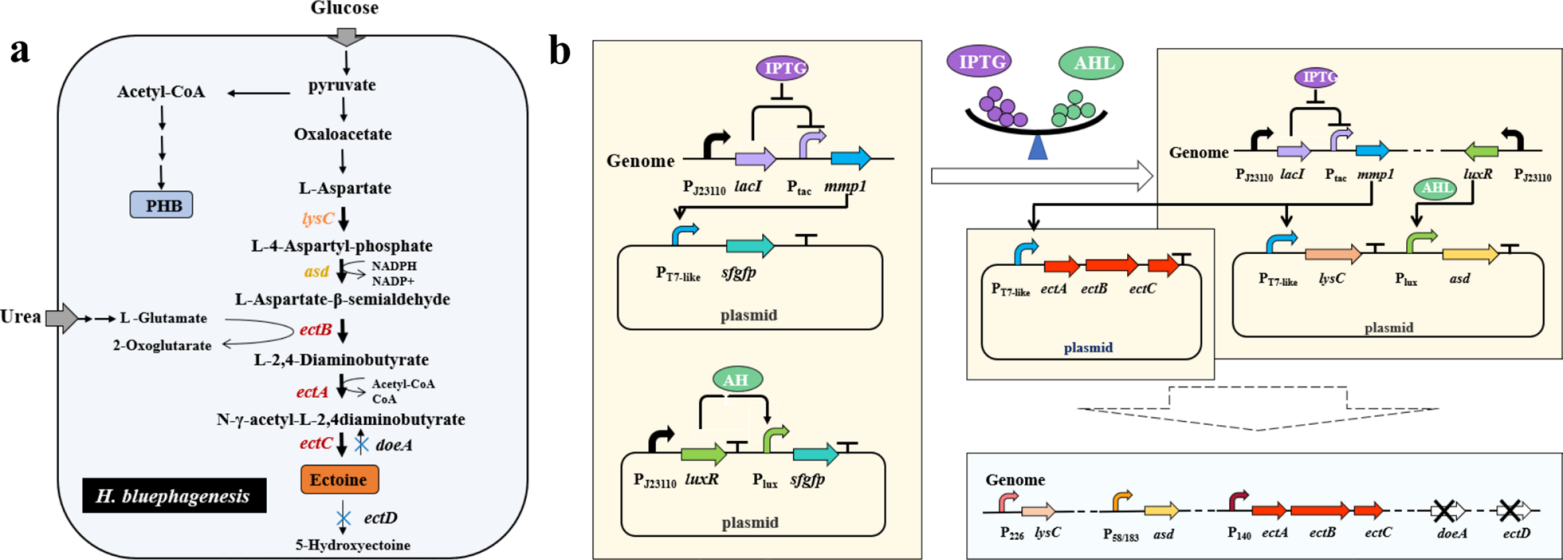
Metabolic engineering modification strategy for ectoine and PHB co-production by *H. bluephagenesis*. **a** The reconstructed ectoine and PHB co-biosynthetic pathway in *H. bluephagenesis*. Modified metabolic fluxes of high efficiency involved in ectoine synthesis are shown in black bold lines. Genes *doeA* and *ectD* encoding ectoine hydrolase and ectoine hydroxylase, respectively, are deleted. **b** Application of two orthogonal inducible systems in ectoine synthesizes related genes. Two orthogonal inducible systems and fine-tuning of *asd*, *lysC* and *ectABC* genes

Recently, Ma et al*.* developed an efficient de novo ectoine biosynthetic pathway by rational flux-tuning of the moderately halophilic *Halomonas bluephagenesis* in an open unsterile fed-batch fermentation (Fig. 3) [36]. They firstly confirmed the function of *ectABC* gene cluster from *H. bluephagenesis* by measuring the ectoine production in the start host *H. bluephagenesis* TD1.0 and the recombinant *E. coli*. Based on its native ectoine biosynthetic system, *H. bluephagenesis* TD1.0 grown in the medium supplemented with 1.03 M NaCl produced 0.63 g/L ectoine for resisting the hyperosmotic stress. To circumvent host’s regulatory system normally controlled by salt concentration and further enhance the production of ectoine, a T7-like plasmid expression system was employed to optimize the expression of *ectABC* in *H. bluephagenesis* TD1.0 and the *doeA* and *ectD* genes were deleted to prevent ectoine from degradation [73]. By adopting these strategies, up to 3.2 g/L ectoine was obtained, representing a great improvement in ectoine production. Given chromosome integration of target genes enables the robust growth of chassis cells in antibiotic free medium, the *ectABC*, *lysC* and *asd* genes were inserted into the *H. bluephagenesis* chromosome for constitutive expression. In addition, the transcription levels of the three isolated gene clusters were fine-tuned using a GFP-based transcriptional tuning method. Finally, the engineered *H. bluephagenesis* TD-ADEL-58 produced 28 g/L ectoine within 28 h in a fed-batch fermentation, with a concomitant production of poly-β-hydroxybutyrate (PHB) inside the cell as a co-product [36]. Therefore, the moderately halophilic *H. bluephagenesis* represents a promising chassis cell for co-production of ectoine and PHB, promoting the development of next generation industrial biotechnology.

## Conclusions

*Halomonas elongata* is currently used as the major bacterium for the commercial production of ectoine. However, the high-salt fermentation broth used for efficient ectoine biosynthesis in this process is corrosive to the fermentation tank and difficult for the downstream wastewater treatment. Therefore, efforts have been made to develop other chassis cells for efficient production of ectoine/hydroxyectoine with lowered salt levels. The engineered model microorganisms such as *E. coli* and *C. glutamicum* are promising for the conventional industrial production of ectoine/hydroxyectoine, as they are capable of producing large amount of ectoine/hydroxyectoine under low salt conditions.

Given ASA is an important precursor molecule in ectoine/hydroxyectoine biosynthetic pathway, most of the model microorganisms employed for the heterologous production of ectoine/hydroxyectoine are good producers of aspartate-derived amino acids, which can provide ectoine/hydroxyectoine biosynthesis with sufficient ASA precursor. However, large amounts of amino acids are produced concomitantly as by-products. Though the disruption of the amino acid biosynthetic pathways is a preferred strategy for high-specificity production of ectoine/hydroxyectoine, the elimination of amino acid production usually results in the decreased production of ectoine/hydroxyectoine. Therefore, prevention of amino acid excretion could be considered as an alternative strategy to simplify the downstream processing of ectoine/hydroxyectoine.

Transcriptional balance has shown to be of great importance in ectoine production. High expression of EctB and high ratio of EctB/EctA are key to the efficient ectoine production, indicating that EctB is a rate-limiting enzyme in ectoine biosynthesis. With the fast development of multi-omics, molecular systems biology and synthetic biology, one might expect more economical production of ectoine and hydroxyectoine in future.

## Data Availability

Not applicable.

## Abbreviations

Ask: L-Aspartate kinase
Asd: L-Aspartate-β-semialdehyde dehydrogenase
EctB: L-2,4-Diaminobutyrate aminotransferase
EctA: L-2,4-Diaminobutyrate acetyltransferase
EctC: Ectoine synthase
EctD: Ectoine hydroxylase
EutABC: Hypothesized enzymes for hydroxyectoine degradation
DoeA: Ectoine hydrolase
DoeB: *N*-α-Acetyl-l-2,4-diaminobutyric acid deacetylase
DoeD: Diaminobutyric acid transaminase
DoeC: Aspartate-semialdehyde dehydrogenase
MSC: Mechanosensitive channels
ASA: Aspartate-β-semialdehyde
Pyc: Pyruvate carboxylase
Hom: Homoserine dehydrogenase
